# Zeolite-based nanocomposite as a smart pH-sensitive nanovehicle for release of xylanase as poultry feed supplement

**DOI:** 10.1038/s41598-021-00688-7

**Published:** 2021-11-01

**Authors:** Fariba Dashtestani, Leila Ma’mani, Farzaneh Jokar, Morteza Maleki, Mohammad Eskandari Fard, Ghasem Hosseini Salekdeh

**Affiliations:** 1 Department of Nanotechnology, Agricultural Biotechnology Research Institute of Iran (ABRII), Agricultural Research Education and Extension Organization (AREEO), Karaj, Iran; 2 Department of Systems and Synthetic Biology, Agricultural Biotechnology Research Institute of Iran (ABRII), Agricultural Research Education and Extension Organization (AREEO), Karaj, Iran; 3 Department of Molecular Sciences, Macquarie University, Sydney, NSW Australia

**Keywords:** Biophysics, Chemical biology, Materials science, Nanoscience and technology

## Abstract

Xylanase improves poultry nutrition by degrading xylan in the cell walls of feed grains and release the entrapped nutrients. However, the application of xylanase as a feed supplement is restricted to its low stability in the environment and gastrointestinal (GI) tract of poultry. To overcome these obstacles, Zeozyme NPs as a smart pH-responsive nanosystem was designed based on xylanase immobilization on zeolitic nanoporous as the major cornerstone that was modified with L-lysine. The immobilized xylanase was followed by encapsulating with a cross-linked CMC-based polymer. Zeozyme NPs was structurally characterized using TEM, SEM, AFM, DLS, TGA and nitrogen adsorption/desorption isotherms at liquid nitrogen temperature. The stability of Zeozyme NPs was evaluated at different temperatures, pH, and in the presence of proteases. Additionally, the release pattern of xylanase was investigated at a digestion model mimicking the GI tract. Xylanase was released selectively at the duodenum and ileum (pH 6–7.1) and remarkably preserved at pH ≤ 6 including proventriculus, gizzard, and crop (pH 1.6–5). The results confirmed that the zeolite equipped with the CMC matrix could enhance the xylanase thermal and pH stability and preserve its activity in the presence of proteases. Moreover, Zeozyme NPs exhibited a smart pH-dependent release of xylanase in an in vitro simulated GI tract.

## Introduction

Enzyme supplements are used widely in poultry diets in an attempt to improve poultry performance, feed consumption, and also minimized environmental pollutions due to the reduced output of excreta^1^. Different enzyme supplements have been developed with respect to their target substrates in feed ingredients. Exogenous xylanase is an example of an enzyme supplement to improve nutritional factors by efficiently decomposing the non-starch polysaccharides (NSP)^2^, specifically xylans found in the cell walls of cereals. Consequently, a high proportion of entrapped energy in the NSP feed matrices would be liberated^3^. Furthermore, xylanases help relieves certain gastrointestinal problems such as decreased villus height, raised levels of pathogenic bacteria in the intestine, unnecessary mucin excretion^4^, and chronic inflammation^5^. They also improve poultry immunity^6^ and also minimize the harmful effects of *Salmonella* Typhimurium infection^7^ or *Clostridium perfringens*^8^.

However, xylanase would be inactivated by various external and internal factors that limited its efficient exploitation in the feed industry^9^. An important one of the external factors is faced during the industrial feed processes (*e.g.* pelleting) via the heat treatments, high pressures, and chemical treatments (such as pH, surfactant, and solvents)^10^ or during the shelf-life via the environmental parameters like sunlight, moisture, and temperature^11^. Moreover, physiological barriers are an example of the internal factors of the inactivation of xylanase^12^.

Whilst the poultry’s crop has a slightly acidic medium, the proventriculus and gizzard media are acidic, and the proximal end of the intestine (duodenum) is slightly acidic that toward the distal part included jejunum, ileum, and colon it becomes neutral to slightly alkaline. Since the optimum pH for most of the exogenous enzymes is between 4–6^13^, it is most likely that the exogenous enzymes would be active in crop or duodenum. Unfortunately, the limited retention time of the feed in the crop restricts the enzyme function. On the other hand, before any function, the exogenous enzymes may be degraded under the harsh acidic conditions of some parts of GI such as crop, proventriculus, and gizzard or by the endogenous proteolytic enzymes such as pepsin and trypsin^14^. Therefore, a convenient poultry feed xylanase must tolerate the physiological barriers imposed by pH, digesta retention time, and internal enzymes within the gastrointestinal (GI) tract.

A smart nanosystem is equipped with a micro-environmentally sensitive group that enables it to release the loaded cargo at a desired time and location when exposed to the given external stimuli, such as temperature, pH, light, redox properties, enzyme activity, electric & magnetic fields, and ultrasound^15^. These structural changes are basically important characteristics of the smart nanosystem, as they provide and facilitate the exploitation of the inherent characteristics of the GI microenvironment^16^. Among them, the nanosystems with the pendant groups that undergo specific physicochemical changes in response to pH changes are deemed “pH-sensitive”. The smart pH-sensitive delivery nanosystems appear to be highly appealing candidates due to the intrinsic differences of the target tissues in terms of the relative acidity^17^.

Carboxymethyl cellulose (CMC), is an anionic linear polymer, water-soluble, and pH-sensitive^18^. CMC is used in the food industry as an emulsion stabilizer, thickener, moisture binder to create desirable textural of food^19^. CMC has been used in drug delivery widely^20^. Therefore, the CMC-based polymer was used as a biocompatible cottage for pH-sensitive release of xylanase in different parts of GI. There are many reports about stabilizing xylanases as feed supplements against external sources^21–23^. However, to the best of our knowledge, albeit with the extensive research efforts, there is no report in the stabilization of xylanase against the internal causes through the GI.

To address these challenges, enzyme immobilization has been exploited to improve the operational stability of enzymes. An efficient enzyme immobilizing in feed industries requires an appropriate support matrix with definite properties such as inertness, biocompatibility, biodegradability, cost-effectiveness, physical strength, stability, renewability, and ability to preserve enzyme activity^24^. Zeolites are a kind of natural and biocompatible nanoporous aluminosilicates with hydroxyl-rich surface functional groups that their properties such as ion adsorption and cation exchange capacity they are ideal for different applications such as enzyme immobilization^25^. Due to their useful and unique properties, using zeolites in different sectors of poultry industries provides significant opportunities for obtaining an improvement in the performance and quality of production as well as the mitigation of environmental pollutions and waste control processes produced by the poultry and swine industries^26^. One kind of natural zeolite material, clinoptilolite, has been widely considered for its positive effects on health, including detoxification, immune response, removal of harmful substances like heavy metals, ammonia, antioxidants, and the general health status. The consumption of clinoptilolite-based products in vivo has increased vastly^27^. For example, zeolite clinoptilolite is applied as an additive to poultry feed that improves the nutrient consumption and growth^28^.

Herein, to mitigate the mentioned issues, a smart nanosystem enabled to efficient release and protecting enzyme activity was designed for providing enough enzyme concentrations for the desirable function at the duodenum, jejunum, and ileum parts of GI. Generally, to design an efficient support–enzyme interaction, the tailored pendant groups of the organic linker can be grafted onto the surface of nanocarriers. It has been reported that amino acids are a suitable candidate as the linker molecules for enzyme immobilization^29^. Therefore, Lys was selected as the pendant organic linker, which as adopted for poultry feed supplementation^30^. Then, xylanase was immobilized onto the surface of Lys modified zeolite (xylanase@Lys-zeolite) to stabilize xylanase against external deactivation sources. Furthermore, to overcome the internal deactivation of xylanase in GI and to improve its pH-controlled release, the xylanase@Lys-zeolite was encapsulated by CMC and chemically cross-linked using glutaraldehyde (denoted as Zeozyme NPs). As a feed supplement, the structural and functional analysis has revealed that Zeozyme NPs as a smart pH-responsive nanosystem could be preserved enzyme stability and activity in the acidic environment of the stomach.

## Results

### Synthesis and characterization of Zeozyme NPs

**Figure 1:**
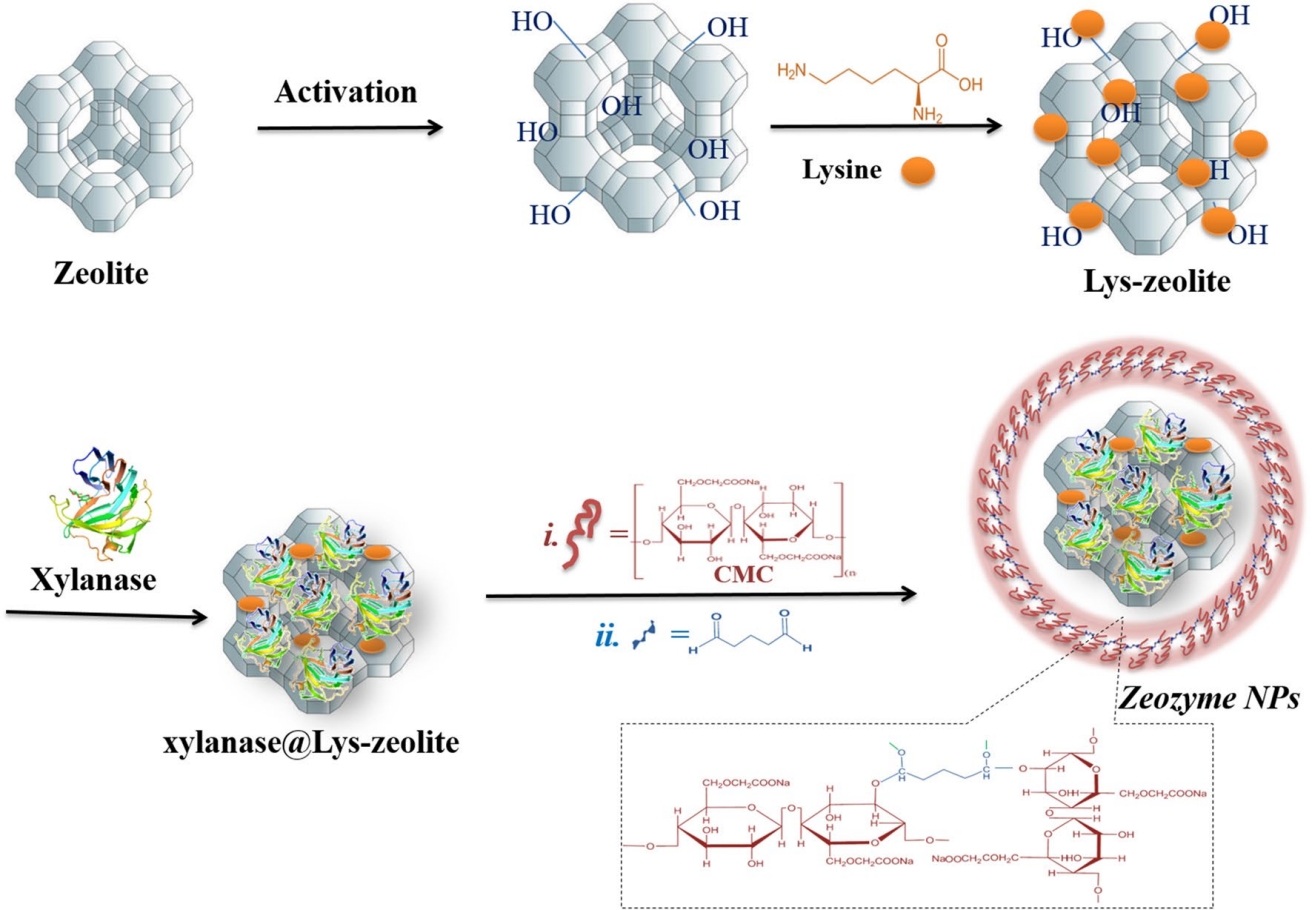
Schematic illustration on the preparation of Zeozyme NPs from zeolite, Lys, xylanase, and CMC polymer as the starting materials.

**Figure 2:**
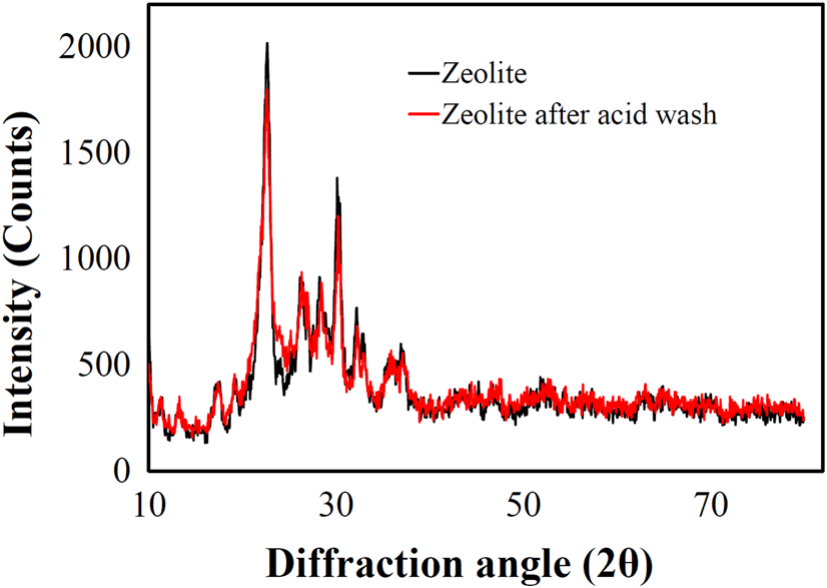
XRD analysis of clinoptilolite zeolite before and after acidic treatment.

The use of exogenous xylanase as a feed supplement would improve poultry performance. However, exogenous xylanase was subjected against two defined deactivation factors: environmental changes (external) and physiological conditions (internal). Consequently, we aimed to introduce the biocompatible Zeozyme NPs programmed for the smart pH-responsive release of xylanase due to the pH changes in GI microenvironment, as a potential feed supplement. The preparation of Zeozyme NPs has been demonstrated in Fig. 1. As illustrated, the nano-encapsulated xylanase called Zeozyme NPs was prepared by immobilizing xylanase onto the nanopores of zeolite (xylanase@Lys-zeolite) and then wrapped by CMC-based polymer. Although the zeolite surface possesses multifunctional groups, these hydroxyl groups interact with each other and form deactivated surface^31^. Hence, the surface of zeolite clinoptilolite particles was activated by refluxing in HCl overnight to break these rings and make reachable hydroxyl groups on the surface. The XRD studies showed that zeolite was not changed after this mild acidic treatment (Fig. 2). In the next step, the zeolite surface was modified by lysine (Lys). The amino acid modified zeolite not only makes a biocompatible surface for xylanase immobilization but also provides more functional groups for the effective binding of xylanase to the surface. Therefore, to achieve a suitable organic linker for improving the physical binding of the enzyme with the rigid surface of zeolite, Lys (an abundant amino acid in poultry feed supplement) were investigated at different weight ratios. The TGA analysis showed that the grafting of Lys was performed more successfully compared to methionine. Therefore, the Lys pendant moieties were applied as a biocompatible and eco-friendly linker for modifying the zeolite surface. Afterwards, xylanase was immobilized onto the surface of Lys-zeolite. The supernatant was examined to determine the enzyme content of the sample. The immobilization efficiency (IE) of Lys-zeolite was calculated by the following equation (Eq. 1), while bovine serum albumin (BSA) was used as standard.

1 $$IE (\%)=\frac{{E}_{i }- {E}_{f}}{{E}_{i}} \times 100$$

where ${E}_{i}$ is the amount of the initial xylanase used in the immobilization and ${E}_{f}$ is the amount of free xylanase detected in the supernatant after centrifugation of the aqueous colloidal solution. The IE for zeolite was 40% while for the Lys modified zeolite (Lys-zeolite), IE was 90%.

**Figure 3:**
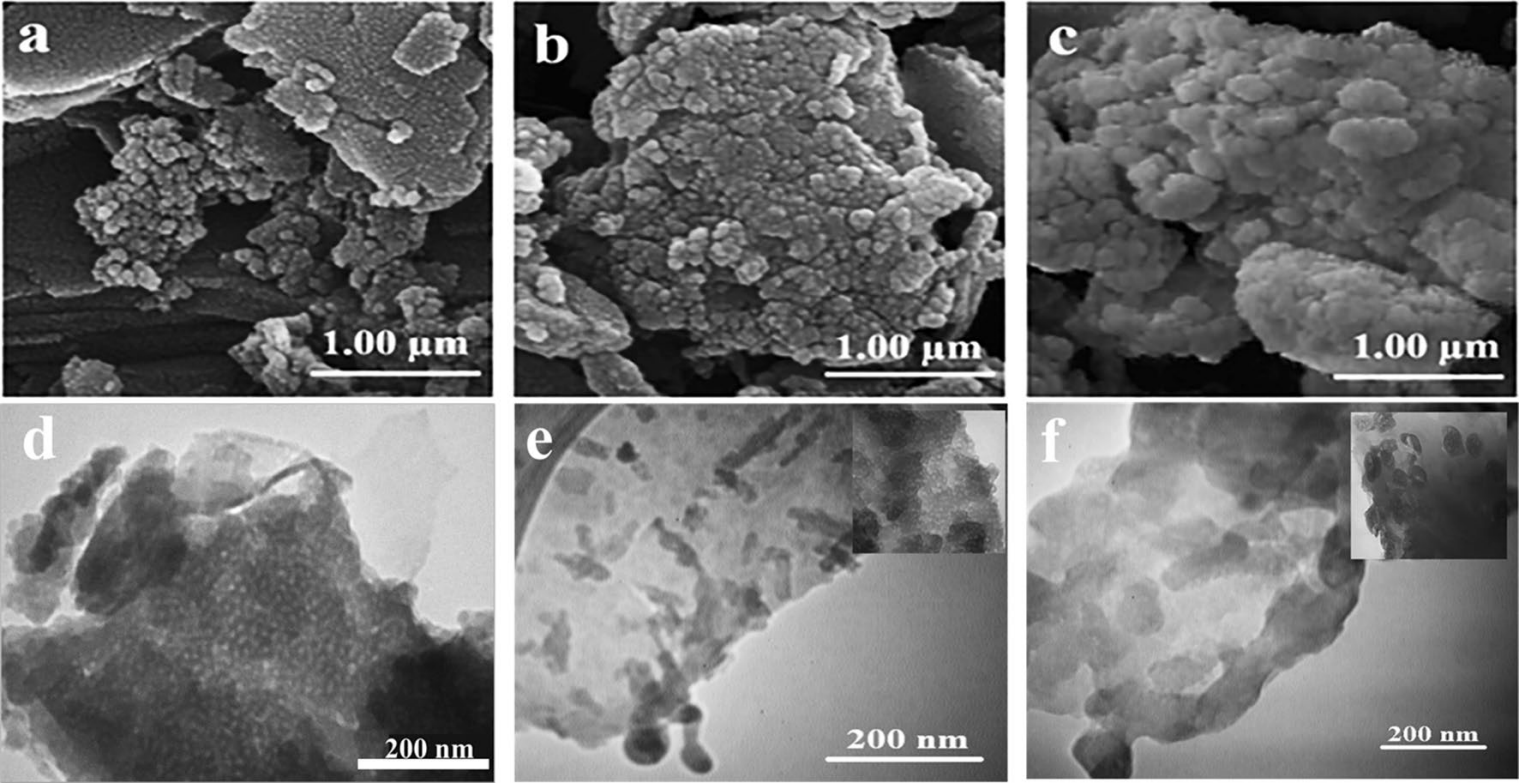
SEM images of zeolite (**a**), xylanase@Lys-zeolite (**b**), and Zeozyme NPs (**c**). TEM images of zeolite (**d**), xylanase@Lys-zeolite (**e**), and Zeozyme NPs (**f**).

Then the xylanase@Lys-zeolite was encapsulated using a glutaraldehyde cross-linked CMC-polymeric shell (Zeozyme NPs) which is capable of preserving the xylanase activity and stability at low pH of the poultry digestion system. One of the common chemical reactions that involves the formation of polymer-based compounds is Schiff base reaction. This reaction comprises of an interaction between two functional groups of amine and aldehyde groups. Some examples of Schiff base reaction involves applying the cross-linking agents^32^. At acidic pH, the cross-linking agent glutaraldehyde can also react with the hydroxyl groups present on the CMC polymer chains (Fig. 1). The glutaraldehyde contains aldehyde groups at the terminal end of the molecule, which react with the hydroxyl groups on the adjacent CMC chains. To roll out the leaching of xylanase during the CMC-encapsulation, the amount of xylanase in the supernatant of this step measured by UV–Vis spectroscopy was zero. The Zeozyme NPs structure was characterized by different techniques such as SEM, TEM, AFM, DLS, BET and BJH, and TGA analyses. The nanoporous network has revealed an ordered array of hexagonal honeycomb faces of pores as visualized using SEM (Fig. 3c). The SEM image of Zeozyme NPs would be compared with their starting materials as zeolite (Fig. 3a), xylanase@Lys-zeolite (Fig. 3b). TEM images of zeolite, xylanase@Lys-zeolite, and Zeozyme NPs were illustrated in Fig. 3d–f. The TEM image showed that Zeozyme NPs are spherical with average diameters below 200 nm (Fig. 3f).

**Figure 4:**
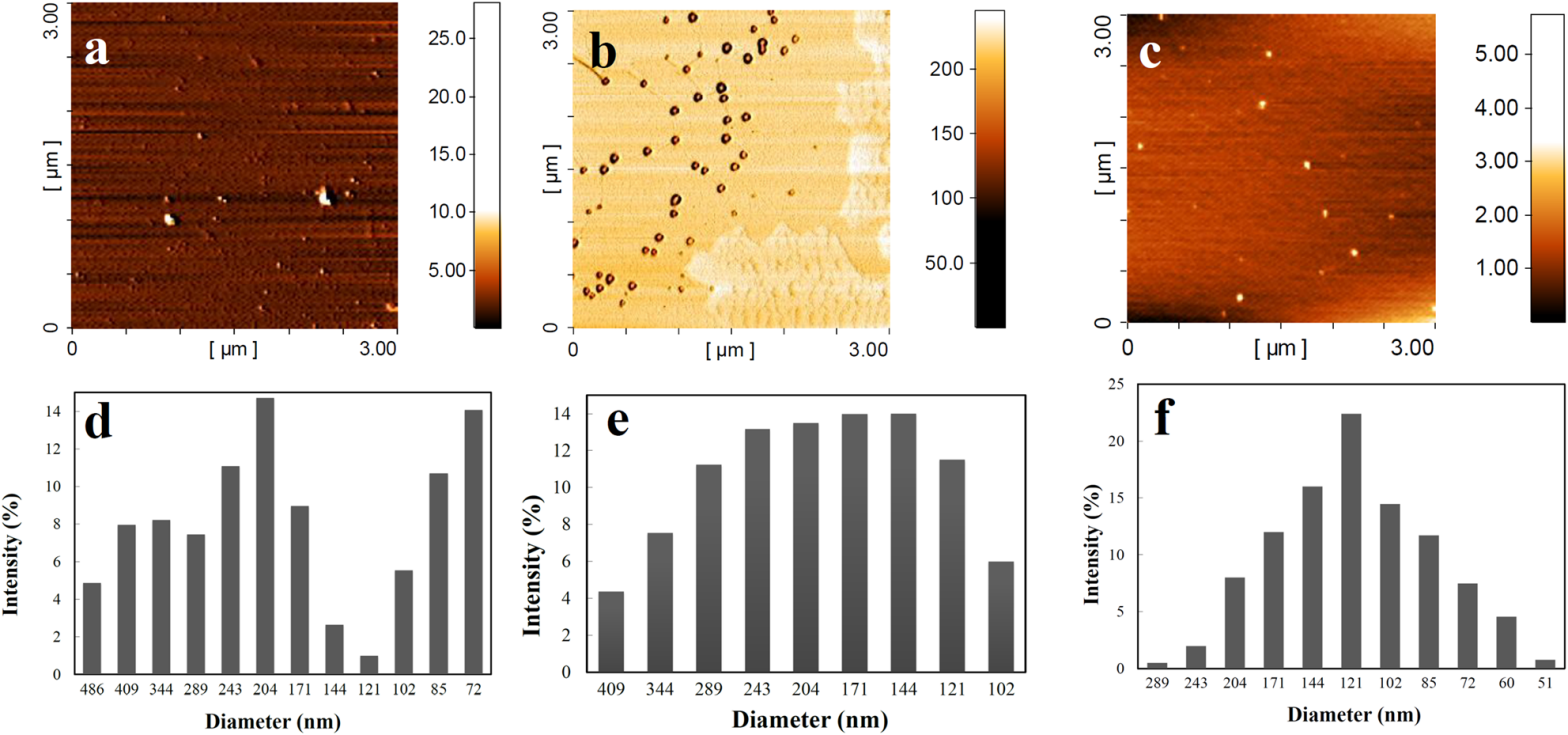
AFM images of zeolite (**a**), xylanase@Lys-zeolite (**b**), and Zeozyme NPs (**c**). Particle size distribution of zeolite (**d**), xylanase@Lys-zeolite (**e**), and Zeozyme NPs (**f**).

The AFM imaging of Zeozyme NPs and the related intermediate materials have been demonstrated in Fig. 4. The morphology of zeolite was diverse and it has revealed that its particles were easily agglomerated, which makes different size ranges of particles (Fig. 4a). However, after immobilization and encapsulation, the uniform morphology with high dispersity was occupied for the xylanase@Lys-zeolite (Fig. 4b) and Zeozyme NPs (Fig. 4c). The particle size of zeolite, xylanase@Lys-zeolite, and Zeozyme NPs was determined using DLS and compared in Fig. 4d, e and f, respectively. As seen, the average diameters of diluted zeolite were slightly larger than that of Zeozyme NPs (800 and 120 nm, respectively). Comparison between DLS data of the starting material and final product revealed that the microscale size of zeolite (800 nm, PDI 0.38) was reduced to the nanoscale and homogeneous nanoparticles of Zeozyme NPs (120 nm, PDI 0.23). Reducing the size from microscale to nanoscale and decreasing the polydispersity is mainly due to encapsulation by CMC. In fact, in this method, immobilized xylanase on zeolite (xylanase@Lys-zeolite) was exposed to CMS that was cross-linked by glutaraldehyde. In such a mixture, CMC polymer nano-network often forms to entrap the xylanase@Lys-zeolite.

The zeta potentials measurement results showed that the charges of zeolite, Lys-zeolite, xylanase@Lys-zeolite, and Zeozyme NPs were − 31, − 22, − 54, and − 25 mV, respectively. This observation suggested that the hydroxyl ions on the surface caused the negative zeta potential of zeolite and upon the surface modifying with positively charged l-lysine amino acid, the zeta potential became a little positive compared to the native zeolite surface. This confirmed that the zeolite surface was modified by l-lysine amino acid. The zeta potential of used xylanase in this research was − 30 mV. The xylanase was immobilized on Lys modified zeolite by covalent binding of amine and carboxylic groups. In this regard, the zeta potential of xylanase@Lys-zeolite reached − 54 mV. The pI of CMC depends on the pK_a_ values of the two functional groups including NH_2_RCOO^−^ and NH_3_^+^ RCOO^−^. This pI is 5.14 and the zeta-potential is negative above the pI due to the larger amount of COO^−^ groups and the zeta-potential is positive below pI due to larger amount of –NH_3_^+^groups^33^. Consequently, the zeta-potential of CMC is positive because of the used acetate buffer (pH 5) for immobilization. As the xylanase@Lys-zeolite (− 54 mV) interacted with CMC the zeta potential of the final product turns − 25 mV. Hence, the zeta potential of Zeozyme NPs was more positive compared to the xylanase@Lys-zeolite, which indicates the loading of enzyme on the surface through the electrostatic interaction.

**Figure 5:**
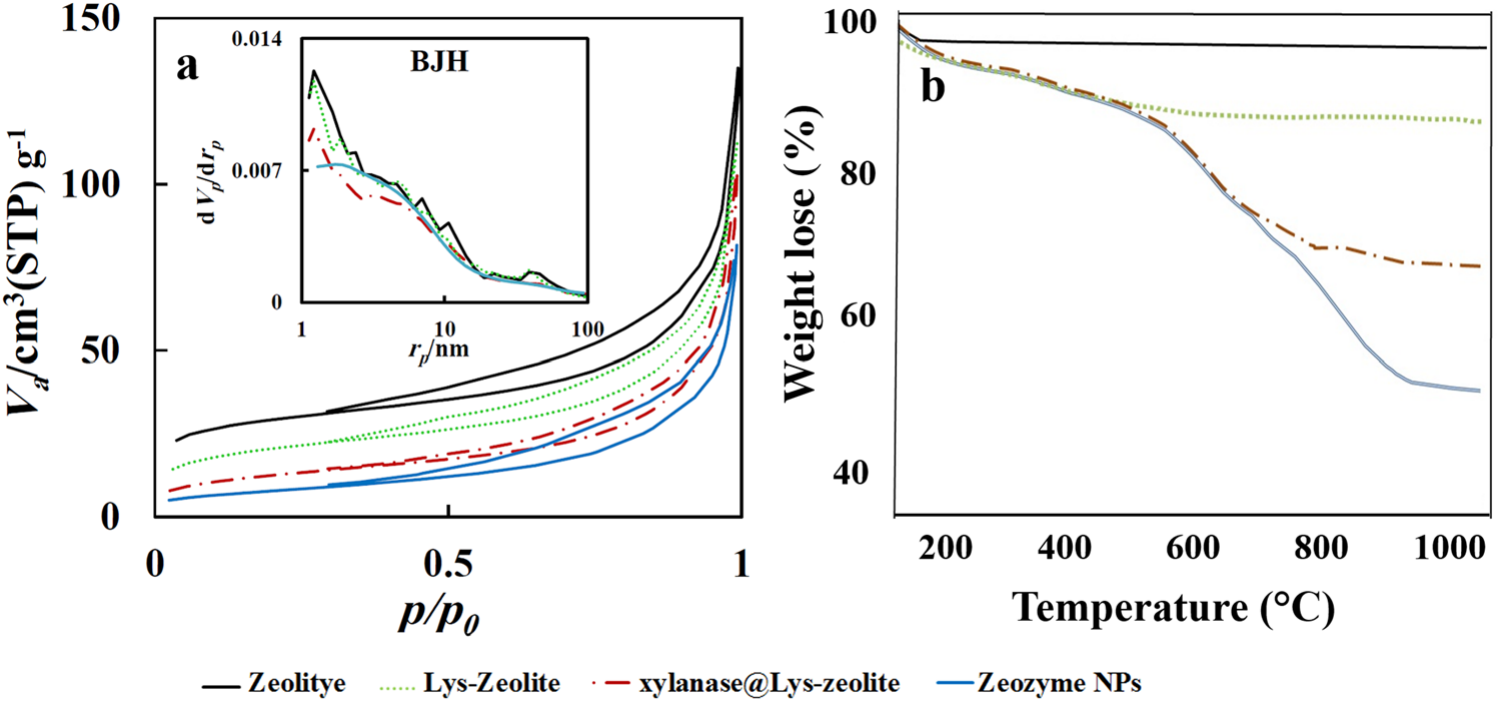
(**a**) The nitrogen sorption isotherm and BJH analysis (inset) of zeolite (dotted line), Lys-zeolite (black solid line), xylanase@Lys-zeolite (dash-dotted line), and Zeozyme NPs (blue solid line). (**b**) TGA analysis of zeolite, Lys-zeolite, xylanase@Lys-zeolite, and Zeozyme NPs.

The N_2_ adsorption–desorption isotherm experiment has shown the pore volume and surface area in the zeolite. The specific surface area S_BET_ of the samples was calculated based on the multiple-point Brunauer–Emmett–Teller (BET) method. As the results of BET analysis summarized in Table 1, a comparison of the data shows that the amount of surface area and pore size of zeolite after xylanase immobilization has decreased. This decrease indicated that the L-lysine, which modified the zeolite, was located both inside and outside surface of the porous network of zeolite. Also, the decreased surface area and pore volume of zeolite after xylanase immobilization confirmed that xylanase was immobilized on the modified zeolite. The nitrogen adsorption–desorption isotherm at 77 K of the zeolite and Zeozyme (Fig. 5) has revealed that the isotherms were of hybrid type I and type IV(a) according to the IUPAC classification^34^ with a H3/H4 hysteresis cycle. Although the 2D layered materials were mostly or fully microporous, some of them showed a mesoporosity as revealed by the hybrid type I and type IV isotherms and the type H3/H4 hysteresis loops^35^.

**Table 1:** The results obtained from BET and BJH of Zeolite and zeozyme NPs.

Material	a_s,BET_ (m^2^ g^−1^)	Total pore volume (cm^3^ g^−1^)	r_p_ (nm)
Zeolite	103.77	0.18	1.93
xylanase@Lys-zeolite	73.965	0.1534	1.21
Zeozyme NPs	28.292	0.1418	3.2

TGA analysis of bare zeolite, Lys-zeolite, xylanase@Lys-zeolite, and Zeozyme NPs are shown in Fig. 5. In all of the samples, the observed weight loss within about 100 °C is related to the elimination of the adsorbed water molecules. The TGA analysis of Zeozyme NPs showed a second peak at about 200 °C, corresponding to the loss of the Lys as the organic linker group. The third peak at 600 °C was related to the loss of xylanase immobilized on the surface of the zeolite. The amount of loaded Lys and glutaraldehyde cross-linked CMC in Zeozyme NPs were determined ~ 10%, and ~ 30%, respectively, as measured using TGA. These results were further confirmed using elemental analysis. Also, the amount of immobilized xylanase onto the zeolite surface measured by thermal analysis was ~ 100 mg xylanase per gram of Zeozyme NPs.

### In vitro evaluating study of zeozyme NPs

#### Activity assay

The function of xylanase was based on degrading of the linear polysaccharide xylan into xylose by catalysis the hydrolysis of the glycosidic linkage (*β*-1,4) of xylosides (Eq. 2). The catalytic activity of xylanase entrapped in xylanase@Lys-zeolite (52 U mL^−1^) and Zeozyme NPs (54 U mL^−1^) were measured and compared with free xylanase (55 U mL^−1^). Any significant difference in the specific activity between free and entrapped xylanase was not observed. It means that immobilization and encapsulation did not affect the catalytic activity. A comparison of the enzymatic activity before and after encapsulation showed litter changes. This is because encapsulation with a polymer makes better dispersity and size reduction. These two parameters are very significant in catalytic activity^36^.2

#### Thermal and pH stability

**Figure 6:**
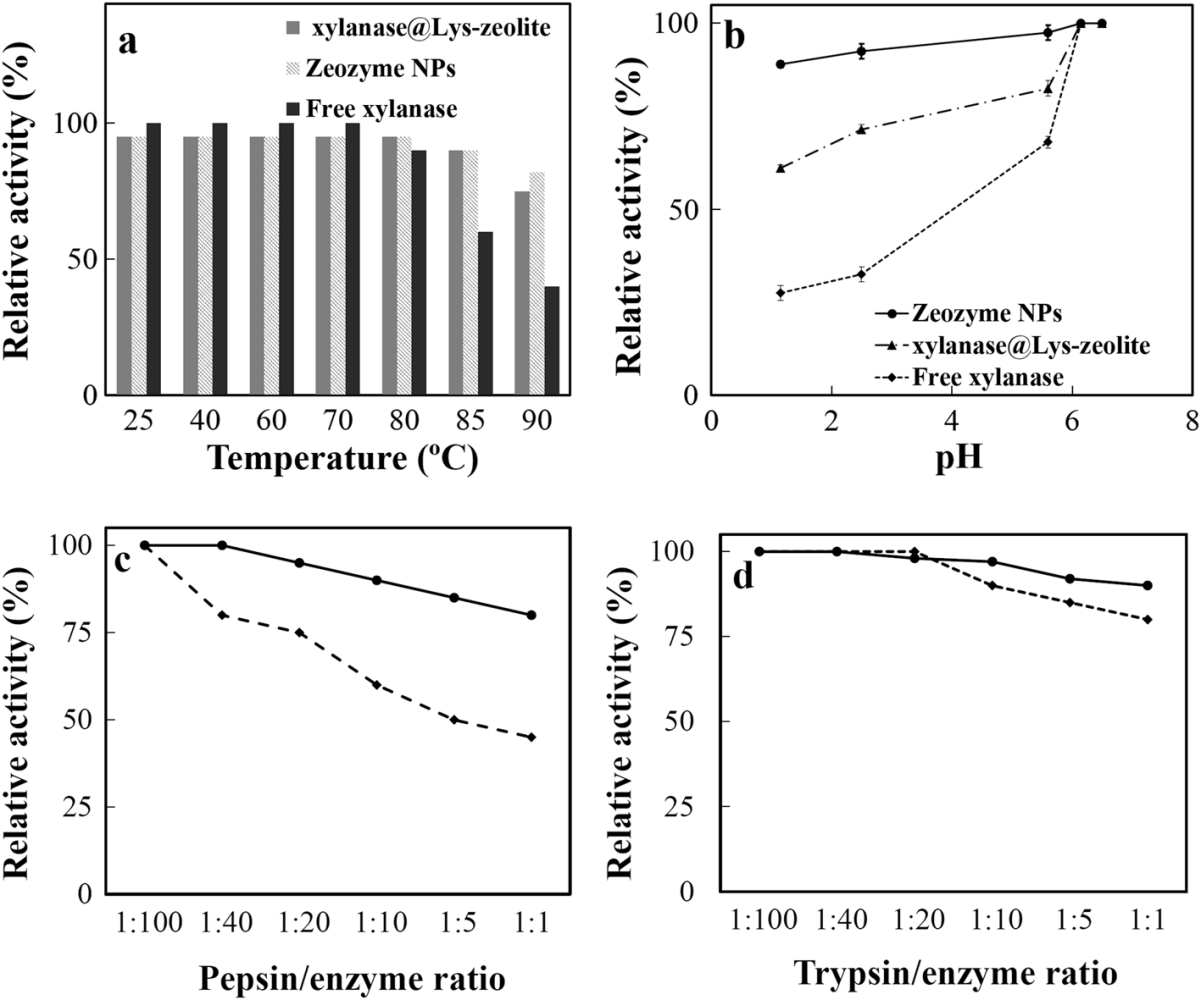
(**a**) The thermal, and (b) pH stability comparison between free xylanase, xylanase@Lys-zeolite, and Zeozyme NPs. Resistance evaluation of xylanase (dotted lines) and Zeozyme NPs (solid lines) to (**c**) pepsin at pH 2.0, and (**c**) trypsin at pH 7.0 at various pepsin or trypsin/enzyme ratios. All samples were incubated at 41 °C for 1 h.

The stability of immobilized xylanase in the form of xylanase@Lys-zeolite and Zeozyme NPs were evaluated against temperature and pH verification. The thermal stability of free xylanase, xylanase@Lys-zeolite, and Zeozyme NPs from 60 to 90 °C for 45 min has been demonstrated in Fig. 6a. The immobilized and encapsulated enzyme was more stable against the thermal treatment than the free enzyme (Fig. 6a). Furthermore, the pH stability of free xylanase, xylanase@Lys-zeolite, and Zeozyme NPs in the pH and retention time simulated GI tract was studied. The samples were incubated in various pH from 1.1 to 7.1 followed by the xylanase activity assay. In acidic pH, free xylanase was deactivated but xylanase@Lys-zeolite and Zeozyme NPs retained hydrolysis activity (Fig. 6b). A Comparison of the pH-stability between xylanase@Lys-zeolite and Zeozyme showed that xylanase@Lys-zeolite preserved about 60% activity in acidic pH compared to its activity in neutral pH, but Zeozyme NPs completely maintained its activity in all of the pH ranges.

This thermal stability of xylanase@Lys-zeolite and Zeozyme NPs would be explained by zeolite role in the immobilization. Zeolite as an electric insulator possesses a low dielectric constant, but it is able to transport heat away from the electric components^37^. For example, the immobilization of lipase^38^ or glucose oxidase^39^ on zeolite enhanced the enzymes’ thermos-stability. Zeolite with a high surface area shows high stability against variation in the temperature, pH and organic solvents^40^. Hence, with regard to these properties zeolite would shield xylanase against thermal and pH changes. In addition, xylanase un-folding was restricted by proper linkage via Lys on the porous surface of zeolite. As a whole, Zeozyme NPs up to 90% and xylanase@Lys-zeolite about 60% can preserve hydrolytic activity of xylanase comparing to free xylanase.

#### Pepsin and trypsin resistance

The free xylanase, xylanase@Lys-zeolite, and Zeozyme NPs varied in the protease resistance over a range of protease/enzyme mass ratios (Fig. 6c). After the treatment with pepsin, the enzyme activity in Zeozyme NPs remained almost unchanged. A slight decrease in Zeozyme NPs activity was observed from the protease: enzyme ratios of 1:100–1:5. In the ratio of 1:1 the Zeozyme NPs activity reduced to 90% of the initial value (Fig. 6c). The pepsin resistance of free xylanase decreased with the increased pepsin/enzyme ratios, retaining 45% of its activity after the pepsin treatment at a ratio of 1:1 (Fig. 6d). These results indicated that Zeozyme NPs is highly resistant to trypsin and pepsin degradation, while the free xylanase is highly sensitive to pepsin and moderately resistant to trypsin. Enzyme immobilization moderately protected enzyme from denaturation in the acidic pH and proteases in gastrointestinal conditions such as phytase immobilized on hydroxyapatite NPs^41^. Thus, zeolite may play a suitable role in the stabilizing the xylanase against pepsin. But, CMC cottage plays a superior role in stabilizing enzyme against acidic environment and pyrolytic enzymes of the digestive system.

#### In vitro* pH-controlled release of xylanase*

**Figure 7:**
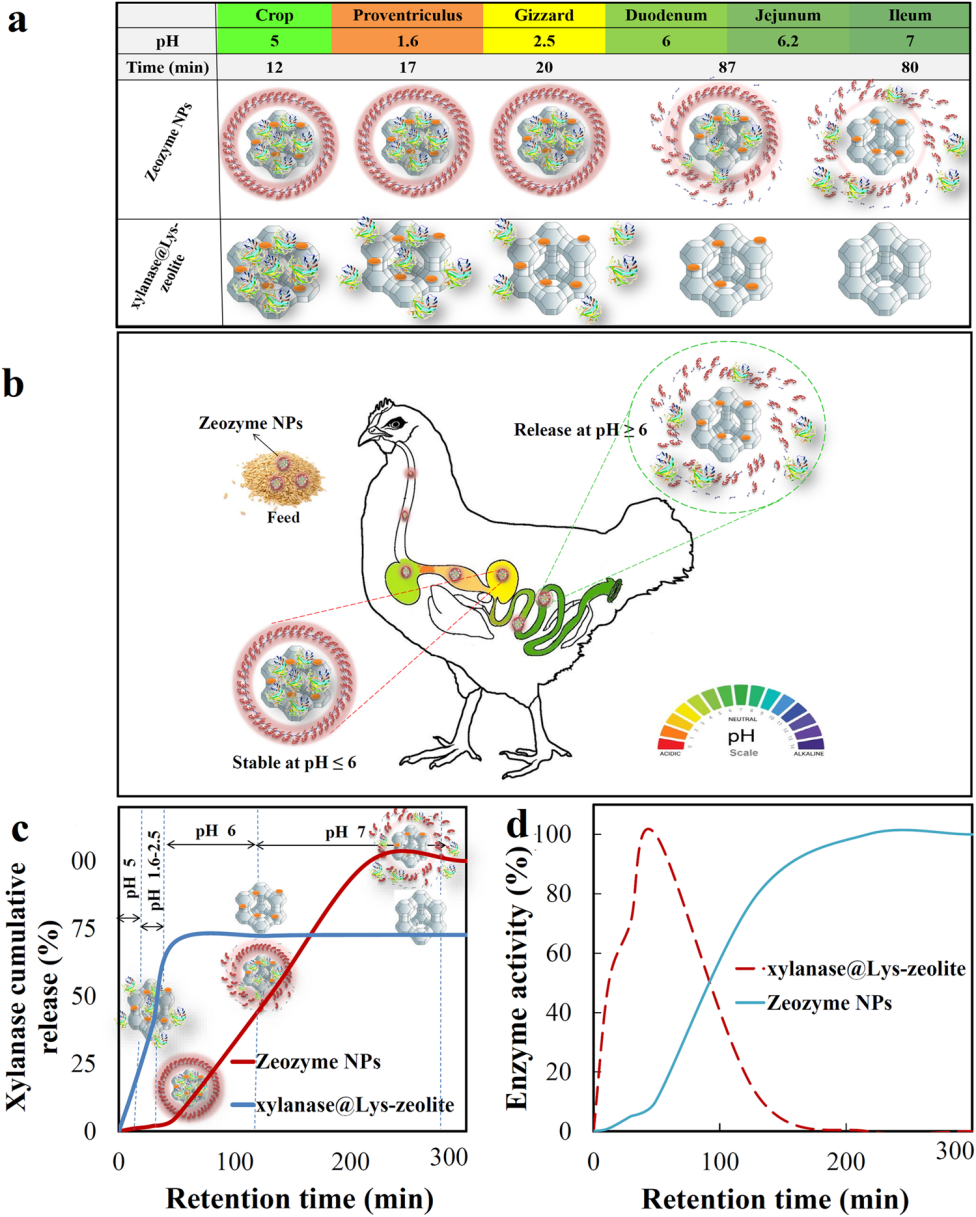
(**a**) Schematic comparison of pattern of release of xylanase from xylanase@Lys-zeolite and Zeozyme NPs in the simulated variable pH conditions and retention time of passage through the GI tract. (**b**) GI tract pH profile of poultry showed by color scale. (**c**) Xylanase cumulative release comparison between xylanase@Lys-zeolite and Zeozyme NPs at 41 °C, where the xylanase release was evaluated by stirring samples at pH 5.2 (for 12 min), pH 1.6 (for 15 min), pH 2.5 (for 20 min), pH 6.0 (for 87 min), and pH 7.1 (for 80 min). (**d**) Enzyme activity of released xylanase comparison between xylanase@Lys-zeolite and Zeozyme NPs at 41 °C based on pH and retention time of GI.

To evaluate the efficacy of Zeozyme NPs as a tailored pH-controlled released system for xylanase delivery in GI tract, the xylanase release from xylanase@Lys-zeolite and Zeozyme NPs were investigated in vitro*.* This evaluation also confirmed the necessity of the presence of CMC polymer cottage in Zeozyme NPs. This implied that the release experiments were performed in one buffer solution, so the concentration of the samples was retained. To simulate GI passage, the samples were tested at pH 5.2 for 12 min (the crop), at pH 1.6 for 17 min (the proventriculus), at pH 2.5 for 20 min (the gizzard), at pH 6.0 for 87 min (the duodenum and jejunum), and at pH 7.1 for 80 min (the ileum). The processes of xylanase release from xylanase@Lys-zeolite and Zeozyme NPs were demonstrated in Fig. 7a. As it showed in the case of xylanase@Lys-zeolite, the most amount of xylanase was released in the forestomach (crop, proventriculus, and gizzard). But in Zeozyme NPs, abundantly were released in the posterior digestive tract (duodenum, jejunum, and ileum). The passage time of feed and the pH value of different parts of the digestive tract of poultry were also illustrated in Fig. 7b by color changes. The passage time of feed in the anterior of the digestive tract of poultry is relatively short and the xylanase could not have enough time to hydrolysis the feed completely. Also, xylanase showed the best activity in neutral pH. Therefore, the best exogenous feed enzyme must be release in the posterior digestive tract where enough time and suitable pH for degrading feed effectively is available. As seen in Fig. 7c, about 50% of xylanase was rapidly released from xylanase@Lys-zeolite in pH 1.1–2.5 during the first hour. In contrast, Zeozyme NPs released less than 4% of xylanase within the first hour and the majority of xylanase (about 95%) was released within 4 h. Figure 7d showed the enzyme activity of released xylanase from xylanase@Lys-zeolite and Zeozyme NPs *in-vitro* under different simulated conditions of GI. The enzyme activity data (Fig. 7d) was in correlation with the amounts of released enzyme in Fig. 7c. This data indicates that the glutaraldehyde cross-linked CMC polymeric coating hinders xylanase release in the acidic pH condition of the stomach. Hence, CMC as a smart response moiety would ensure the safe delivery of high xylanase concentration at the target site (duodenum, jejunum, and ileum), minimizing xylanase release in the stomach, and subsequently promote the poultry growth. Thus, Zeozyme NPs may be a promising platform for xylanase delivery that is triggered by pH.

## Discussion

The results have revealed that the Zeozyme NPs containing zeolite, Lys, and CMC-based nanocage increased xylanase stability against the external deactivating factors arisen from environmental changes, industrial feed processes (e.g. pelleting), or the shelf-life conditions. Moreover, to validate the merits of Zeozyme NPs, its stability was assessed under different deactivation factors using mimicked physiological conditions of GI tract. The Zeozyme showed a smart pH-dependent release of xylanase in different sections of the simulated poultry GI tract. This fantastic stability of xylanase would be clarified the intelligent application of a combined stabilization method (included immobilization and encapsulation) of the enzyme for preparing of Zeozyme NPs. Xylanase immobilization onto the surface of zeolite nanonetwork via Lys as an applicable organic linker has made it robust against the harsh external deactivation factors such as varying ranges of pH and temperature. The distinctive physicochemical properties of zeolites and Lys modified zeolites including hydroxyl rich surface, thermo-stability, and biocompatibility make it a potential candidate for enzyme immobilization to improve its function and stability.

Furthermore, CMC polymer as a hydrophilic matrix and pH-sensitive system has offered the physiological stability for xylanase enzyme against internal deactivation such as pyrolytic internal enzymes of the digestive tract and provided a tailored controlled xylanase release, especially in the neutral and alkaline pH. Since the digestions of non-starch polysaccharides take place through the last sections of the poultry GI tract (duodenum, jejunum, and ileum) which is alkaline, the polymeric shield retained the xylanase in the harsh acidic parts of the digestive tract. In addition, the polymeric nanocage system preserved xylanase against the pyrolytic digestion enzyme secreted in the GI tract. Considering the useful components of Zeozyme NPs including zeolite, Lys, and CMC as biocompatible and poultry feed supplementations the Zeozyme NPs would efficiently be applied as a supplementation in feed and promote the poultry industry. The development of biodegradable and smart delivery nanosystems for improving the efficacy of enzyme-based supplementations in the animal feed industry is worth to be investigated in vivo in future studies.

## Experimental section

### Materials and apparatus

A specific natural zeolite, called clinoptilolite, has been purchased from Afrand Toska Company (Iran). It has been approved by EU (70/524/EEC) and FDA (21 CFR 582–2727) to apply in animal feed. l-lysine (Lys), carboxymethyl cellulose sodium (CMC), potassium phosphate buffer, and acetate buffer were purchased from Merck. DNS (3,5-di-nitrosalcylic acid), sodium potassium tartrate, xylan, and xylose were obtained from Sigma-Aldrich. Deionized water was used in all solutions preparation. The particle size distribution and surface charge of samples were achieved by a dynamic light scattering instrument (DLS, Brookhaven, USA) and nano-sizer (Zeta sizer Nano ZS90, Malvern, UK), respectively. The morphology and size of NPs were studied using, scanning electron microscopy (SEM, Hitachi S-4800 II—Japan) and transmission electron microscopy (TEM, Hitachi H-7650, 80 kV—Japan). Nitrogen sorption isotherms of NPs were taken using a BELSORP mini-II (Microtrac Bel Corp Company, Japon) apparatus at liquid nitrogen temperature (77 K), as a volumetric adsorption measurement instrument. To measure the specific surface area, the Brunauer– Emmett–Teller (BET) theory and for calculating the pore-size distributions the Barrett–Joyner–Halenda (BJH) theory were applied. The thermal properties of NPs were accomplished using thermogravimetric analyses (TGA, TA Instrument; model SDT Q600) from 25 to 600 °C (by a rate of 10 °C min^−1^). The topological characteristics of materials were observed using atomic force microscopy (AFM, DME-Ds95-50—Denmark) in ambient conditions at room temperature. The optical measurements were performed using a UV–Vis double-beam spectrophotometer (UV- 3100 PC, Shimadzu).

### Preparation of zeozyme NPs

Firstly, acidic activation of zeolite clinoptilolite surface was done by refluxing 2.0 g of zeolite with hydrochloric acid (1%) overnight. Then it was washed and centrifuged (6000 rpm, 5 min) until the neutral pH. After that, the surface of zeolite was modified by Lys. Briefly, the zeolite (100 mg) and Lys (50 mM) were dispersed by use of sonication in acetate buffer (2 mL, pH 5.0) and the colloidal solution was stirred for 5 h at room temperature. Next, it was centrifuged at 6000 rpm for 5 min, and the solid phase was separated and washed as Lys modified zeolite (Lys-zeolite).

Afterwards, a previously reported thermostable xylanase^25^ was immobilized by mixing 100 mg of Lys-zeolite with 2.5 mL of xylanase solution (5 mg mL^−1^) and allowed to stir overnight at 4 °C in phosphate buffer (20 mM, pH 7.5). Then the mixture was centrifuged (6000 rpm, 5 min) to collect the residual solid. The xylanase@Lys-zeolite was washed twice with the phosphate buffer to eliminate any non-immobilized xylanase. The supernatant was gathered to determine the enzyme content of the sample. The enzyme concentration at first was identified by monitoring the protein specific adsorption peak at 280 nm Nano-drop UV. Then, by subtracting the enzyme concentration before immobilization and remained in supernatant after immobilization the amount of immobilized xylanase would be obtained.

Finally, a clear solution of 75 mg of CMC in 5 mL of deionized water was added to a mixture of 100 mg of xylanase@Lys-zeolite in 2.5 mL of deionized water and then it was left for 20 min at room temperature. After that, glutaraldehyde 4% was poured into the mixture dropwise, and stirred for 2 h at room temperature. In the next step to obtain CMC-based polymer encapsulated xylanase@Lys-zeolite (Zeozyme NPs), the solid residue was separated by centrifugation and it was then washed thoroughly with water and phosphate buffer.

### Loading efficiency

Xylanase concentration entrapped in zeolitic-based nanocarriers was quantified by the Bradford method^42^. Briefly, into a 96-well titer plate, the resulting supernatant (may contain xylanase) (5 *μ*L) and then 245 *μ*L of Bradford reagent were added. The blended sample and Bradford reagent were incubated at 22 °C for 10 min. Subsequently, the absorbance of solution was recorded at 595 nm, and the concentration of xylanase was assessed via a bovine serum albumin-based Bradford standard curve.

Additionally, the stability of xylanase after loading was performed. The xylanase@Lys-zeolite was re-suspended and stirred in a phosphate buffer pH 7 for 2 h. Then the sample was centrifuged (6000 rpm, 5 min). The supernatant was collected and the xylanase concentration was evaluated using the Bradford method to assay the amount of xylanase leaches out from zeolite surface.

### Enzymatic activity assay

Xylanase activity was measured based on Bailey, et al.^43^. Briefly, birchwood xylan solution (900 μL, 1%) as the substrate was added to the xylanase sample and incubated at 50 °C for 30 min. 1.5 mL DNS reagent was added and boiled for 5 min in a water bath to stop the reaction. Afterward, the absorbance intensity at 540 nm was recorded. The calibration standard graph for different concentrations of xylose (0–1 mg) was obtained. One unit of xylanase activity was equal to the amount of enzyme that liberated 1.0 μmol of xylose product per minute under the specified conditions of the assay method as described by Bailey et al.^43^.

### Temperature and pH stability of xylanase

The thermal stability of free xylanase, xylanase@Lys-zeolite, and Zeozyme NPs was assayed after incubating samples separately in a water bath for 60 min at different temperatures (from 30 to 90 °C in phosphate buffer, 10 mM, and pH 7.0). The Effects of pH changes on the enzymatic activity of xylanase were measured and the results were compared for all of the enzyme samples (free xylanase, xylanase@Lys-zeolite, and Zeozyme NPs). The phosphate/acetate buffer was used at 10 mM for different pH values. The xylanase@Lys-zeolite and Zeozyme NPs samples were incubated at the specific temperature or pH, then the samples were centrifuged (at 6000 rpm, 5 min) and the pellet re-suspended in phosphate buffer, (10 mM, and pH 7.0). For free xylanase sample, the pH of buffer was just adjusted to phosphate buffer (pH 7.0). Then all the samples were assayed for evaluating the efficiency of immobilization or encapsulation.

### Pepsin and trypsin resistance

The proteases resistance of enzyme samples (free xylanase, xylanase@Lys-zeolite, and Zeozyme NPs) was evaluated by incubating amount of 40 µg of each sample with various proteases (1:1, 1:5, 1:10, 1:20, and 1:40) at 41 °C for 2 h, and then their xylanase activities were assayed. The examined proteases were pepsin in a glycine–HCl buffer (0.25 M, pH 2.0) or trypsin in a Tris–HCl buffer (0.25 M, pH 7.0)^44^.

### pH-responsive release assay

The assessment of pH-controlled release of xylanase from xylanase@Lys-zeolite and Zeozyme NPs was performed using the corresponded stimulated standard buffers. The buffer acetate/phosphate was prepared by mixing H_3_PO_4_ (0.05 M) and CH_3_COOH (0.05 M) in a ratio of 1:1 (v/v). For designing a model of pH ranges in different parts of GI tract (from 1.6 to 7.1) the prepared buffer was titrated with NaOH (8 M). At the initial point of the experiment, the xylanase amounts of the used samples are the same. The first experiment was done in pH 5.2 and retention time of 12 min as the same as the pH and retention time of feed within the crop. And then, the experiment followed by pH 1.1 to 7.1 by titration with NaOH (8 M). Briefly, xylanase@Lys-zeolite (3 mg) or Zeozyme NPs (10 mg) which possess the same concentration of xylanase (5 mg mL^−1^) were added to a solution (5 mL, pH 5.2) of H_3_PO_4_:CH_3_COOH buffer and put in an incubator shaker for a definite time at 41 °C at 100 rpm for 12 min. Then, the samples were evaluated in the prepared simulated GI medium.

To this, firstly, the buffer solution pH 5.2 for 12 min (the crop) was prepared. After that, a solution with pH 1.6 (simulated pH of the proventriculus for 17 min) was applied and after followed by using NaOH solution to adjust pH to 2.5 (simulated pH of the gizzard for 20 min), to pH 6 (simulated pH of the duodenum and jejunum for 87 min), to pH 6.2–7.1 (simulated pH of the ileum for 80 min). For sampling, 500 µL of solution was removed at specific periods of time and immediately substituted for an equal volume of fresh buffer solution of equivalent pH to maintain a constant volume. The removed samples were centrifuged in 5000 rpm for 5 min and the concentration of xylanase was assayed by Bradford method.
